# Supplementary material for: The Adaptive Value of Chromosomal Inversions and Climatic Change—Studies on the Natural Populations of Drosophila subobscura from the Balkans
Source: Insects. 2023 Jul 1;14(7):596. doi: 10.3390/insects14070596 (PMC10380441; doi:10.3390/insects14070596)
Supplement: Supplementary file 1 [file insects-14-00596-s001.zip › insects-2466924-supplementary.pdf]

Supplementary Materials:

Figure S1. PCoA analysis using the O chromosome inversion polymorphism from Balkan populations. Mt. Parnes (Grece) and Font Groga (Barcelona, Spain) populations were used as reference outgroups. Colors and numbers are the same as in Figure 3.

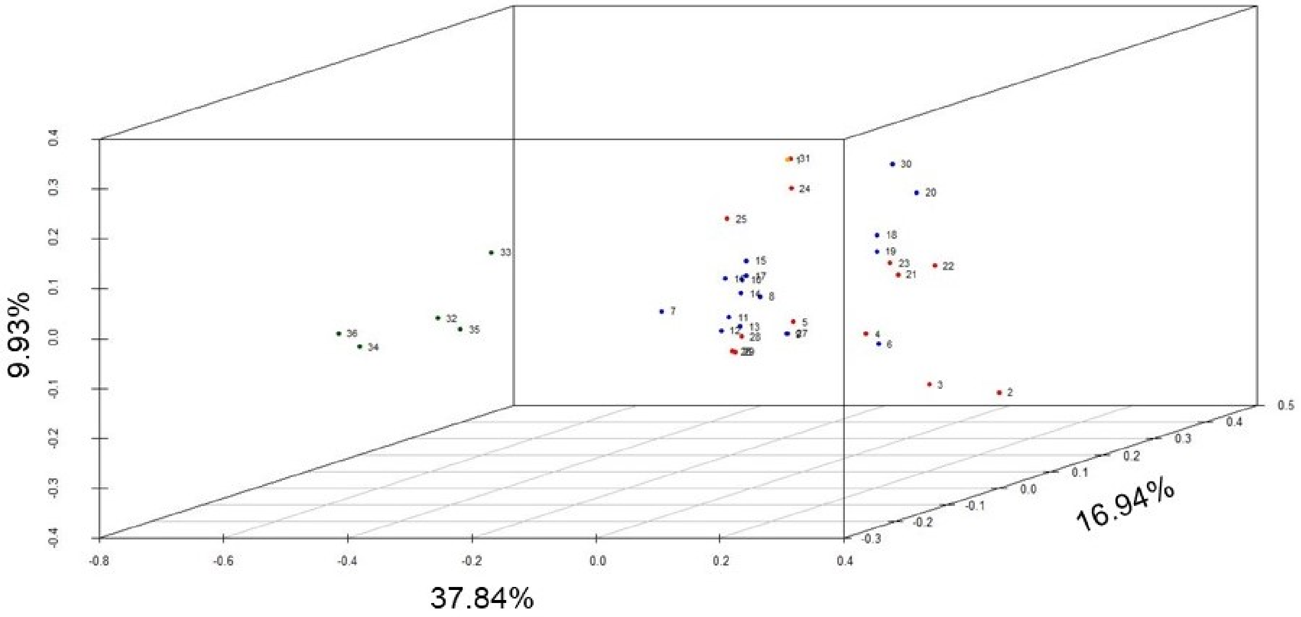

Figure S2. Variation over time (1995-2022) of Tmean (mean temperature), Tmax (maximum temperature), Tmin (minimum temperature), Humidity and Rainfall for the Petnica population. Temperatures were registered in centigrade degrees, humidity in percentage and rainfall in millimeters of precipitation. The slope of the line trend and associated *p* value are also presented.

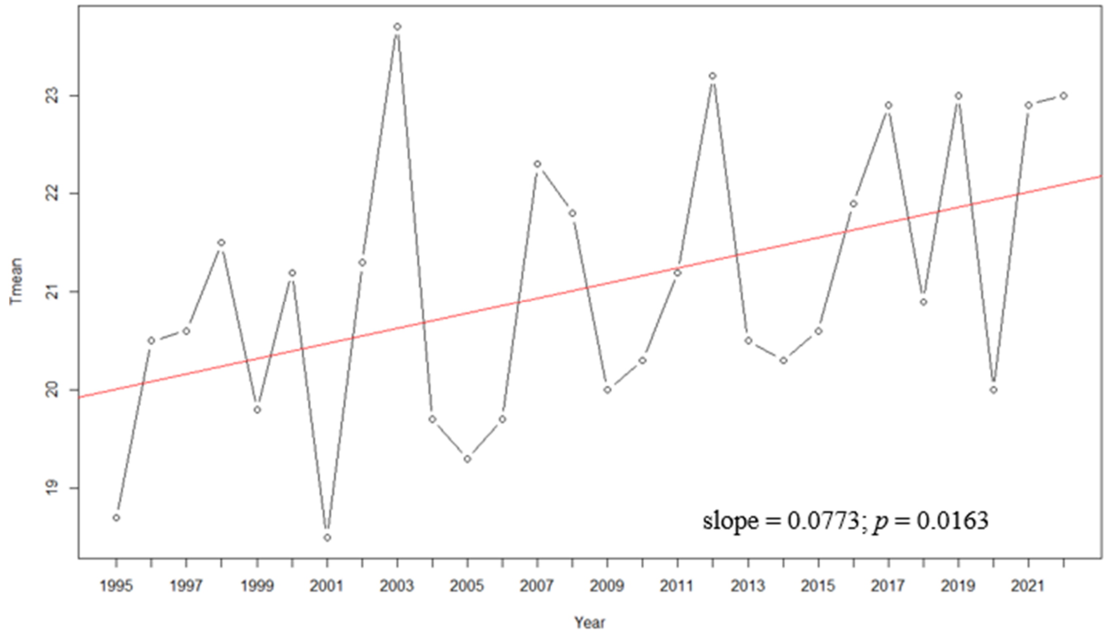

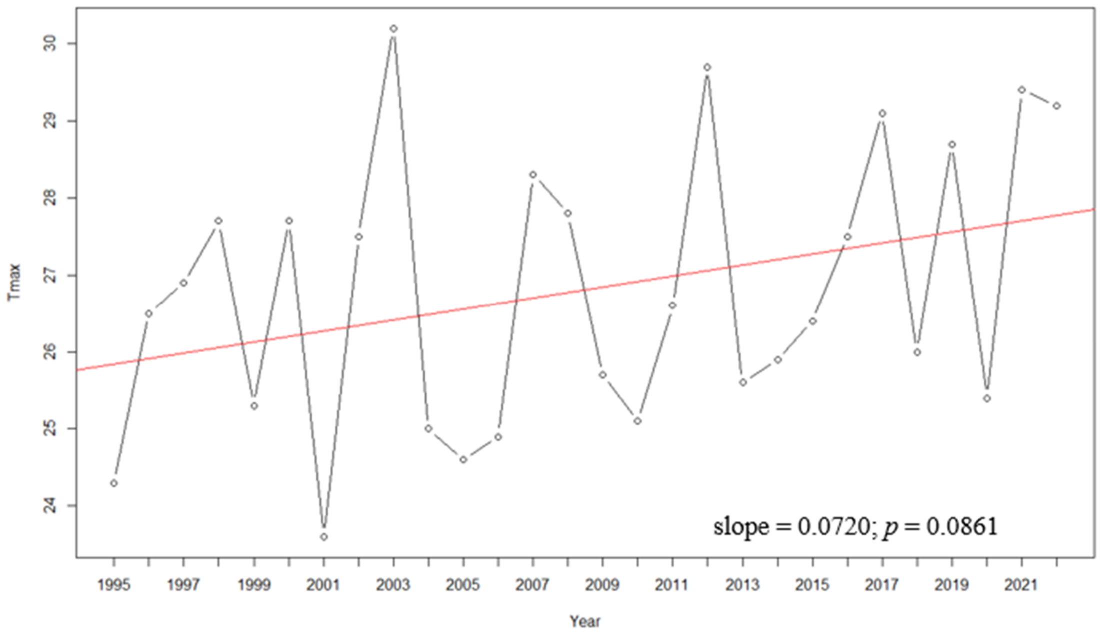

Tmax
30
29
28
27
26
25
24
slope = 0.0720; p = 0.0861
1995
1997
1999
2001
2003
2005
2007
2009
2011
2013
2015
2017
2019
2021
Year

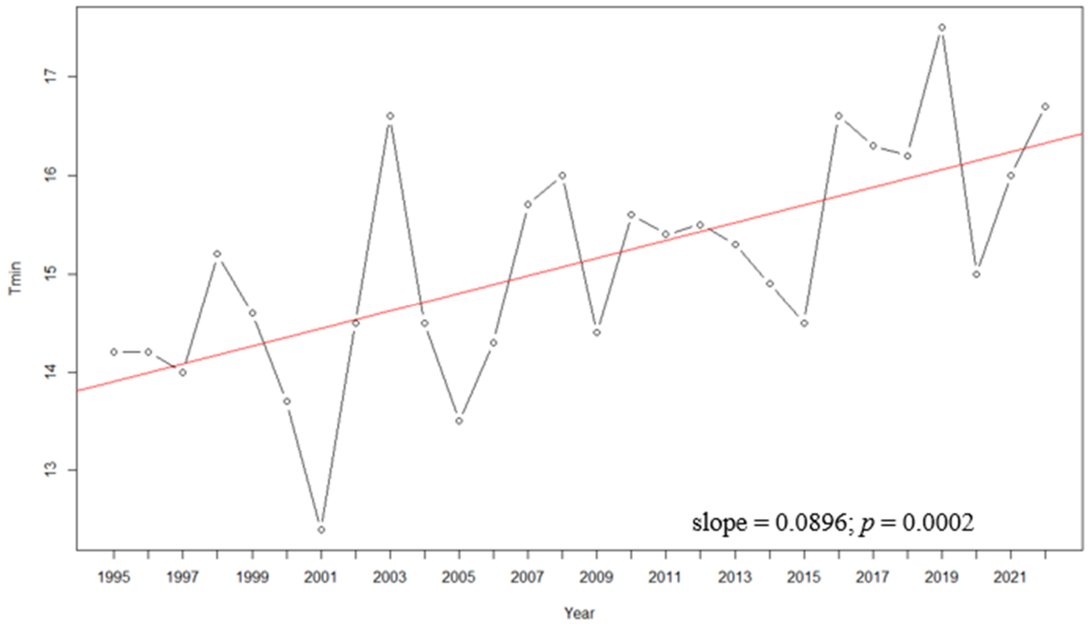

Tmin
17
16
15
14
13
slope = 0.0896; p = 0.0002
1995
1997
1999
2001
2003
2005
2007
2009
2011
2013
2015
2017
2019
2021
Year

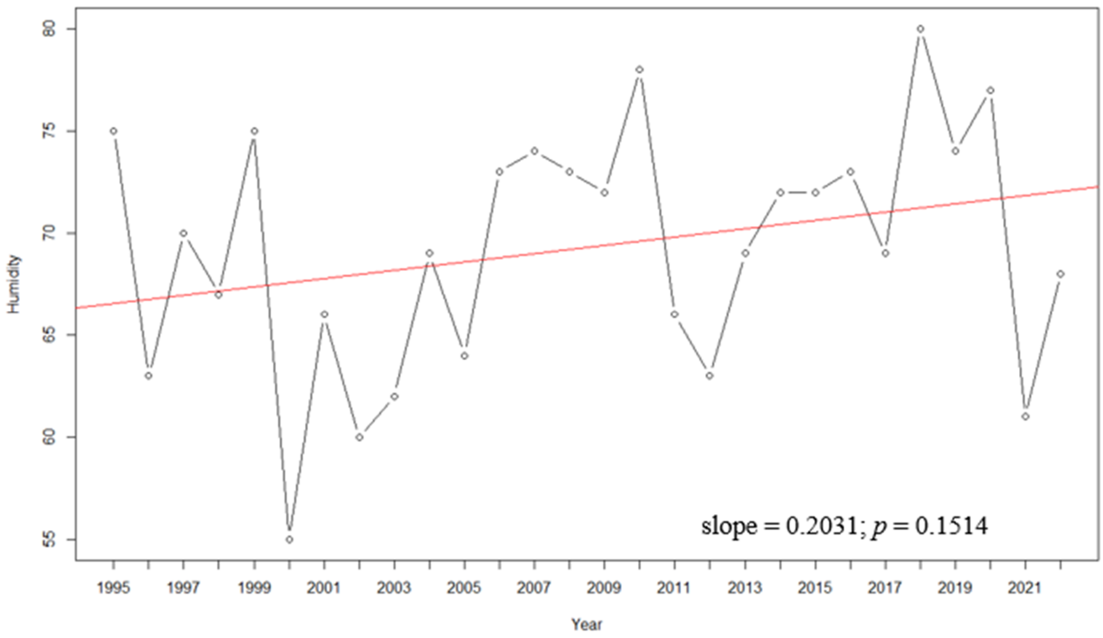

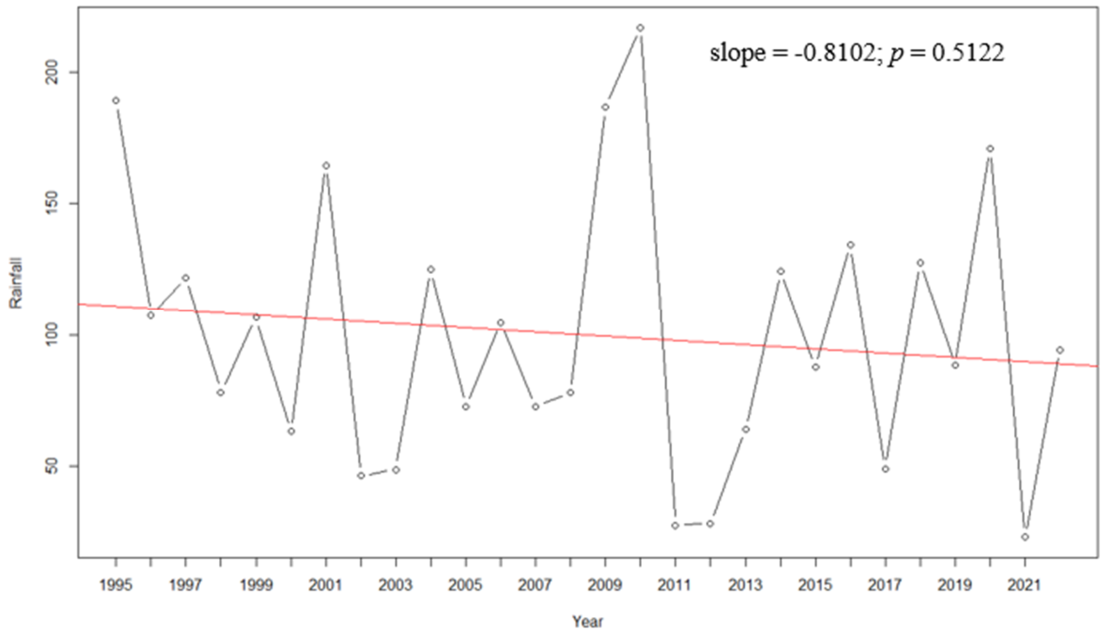

Table S1. The mean of the values obtained for the climatic variables (mean, minimum and maximum temperatures, mean humidity and rainfall) of Petnica during March, April and May for the studied period (2019–2022).

Year	Tmean	Tmin	Tmax	Mean Humid.	Rainfall
2019	12.5	7.2	18.6	70.7	94.9
2020	11.9	6.1	18.3	67.7	46.4
2021	10.7	5.1	16.7	71.7	57.4
2022	11.9	5.4	18.4	66.0	33.6

Units: temperatures are in ºC, rainfall in mm, and humidity in percentage.

Table S2. Statistical study of Hardy-Weinberg equilibrium deviations for J, U, E and O autosomes in years 2019-2022. The values of p and adjusted p, after using the FDR procedure, are shown. Significant values are in bold.

	CHROMOSOMES							
YEAR	J		U		E		O	
	p	*p adj.*	*p*	*p adj.*	*p*	*p adj.*	*p*	*p adj.*
2019	0.8931	1	1	1	0.8062	1	0.995	1
2020	0.6913	0.9217	0.3646	0.8272	0.4136	0.8272	0.9730	0.9730
2021	0.9870	0.9870	0.2977	0.7952	0.3976	0.7952	0.7672	0.9870
2022	0.4915	0.6553	0.1429	0.2857	0.7133	0.7133	**0.0050**	**0.0200**

Table S3. Comparisons between the chromosomal inversion polymorphism of Petnica in 1995 with those from the same population, but for the samples of years 2019, 2020, 2021 and 2022. The values of *p* and adjusted *p* (*p adj*), after using the FDR procedure, are shown. Significant values are in bold.

**Chromosome**	**1995 *vs.* 2019**		**1995 *vs.* 2020**		**1995 *vs.* 2021**		**1995 *vs.* 2022**	
	p	***p adj***	***p***	*p adj*	*p*	*p adj*	*p*	*p adj*
A	0.8052	0.8052	0.2138	0.4276	0.3467	0.4623	0.0959	0.3836
J	**0.0110**	**0.0440**	0.1209	0.1738	0.1499	0.1738	0.1738	0.1738
U	**0.0010**	**0.0013**	**0.0010**	**0.0013**	**0.0010**	**0.0013**	**0.0050**	**0.0050**
E	**0.0040**	**0.0080**	**0.0130**	**0.0173**	0.2488	0.2488	**0.0010**	**0.0040**
O	0.1039	0.1039	**0.0420**	0.0560	**0.0400**	0.0560	**0.0100**	**0.0400**

Table S4. Comparisons between years (1995, 2019 and 2022) for all chromosomes (A, J, U, E and O), considering the thermal adaptations composition of inversions ('cold', 'warm' and 'non-thermal' adapted) for each year in Petnica population. The values of *p* and adjusted *p* after using the FDR procedure are presented. Significant values are in bold.

**COLD INV.**	***p***	***Adjusted p***
A chromosome		
1995 *vs.* 2019	0.8939	0.8339
1995 *vs.* 2022	0.2109	0.3163
2019 *vs.* 2022	**0.0246**	0.0737
J chromosome		
1995 *vs.* 2019	**0.0087**	**0.0261**
1995 *vs.* 2022	0.1523	0.1523
2019 *vs.* 2022	0.6089	0.1339
U chromosome		
1995 *vs.* 2019	0.3369	0.5053
1995 *vs.* 2022	0.2236	0.5053
2019 *vs.* 2022	0.8205	0.8205
E chromosome		
1995 *vs.* 2019	0.0732	0.1098
1995 *vs.* 2022	**0.0030**	**0.0089**
2019 *vs.* 2022	0.1676	0.1676
O chromosome		
1995 *vs.* 2019	0.2107	0.3160
1995 *vs.* 2022	0.1470	0.3160
2019 *vs.* 2022	0.8565	0.8565
**WARM INV.**	*P*	*Adjusted P*
A chromosome		
1995 *vs.* 2019	0.8339	0.8339

1995 *vs*. 2022	0.2109	0.3163
2019 *vs*. 2022	**0.0246**	0.0737
J chromosome		
1995 *vs*. 2019	**0.0028**	**0.0085**
1995 *vs*. 2022	0.0907	0.0907
2019 *vs*. 2022	0.0758	0.0907
U chromosome		
1995 *vs*. 2019	**0.0001**	**0.0003**
1995 *vs*. 2022	**0.0163**	**0.0244**
2019 *vs*. 2022	**0.0312**	**0.0312**
E chromosome		
1995 *vs*. 2019	0.4650	0.4650
1995 *vs*. 2022	**0.0007**	**0.0011**
2019 *vs*. 2022	**0.0007**	**0.0011**
O chromosome		
1995 *vs*. 2019	0.3451	0.5176
1995 *vs*. 2022	0.3031	0.5176
2019 *vs*. 2022	1.0000	1.0000
**NON-THERMAL INV.**	*P*	*Adjusted P*
J chromosome		
1995 *vs*. 2019	0.3215	0.7058
1995 *vs*. 2022	0.5731	0.7058
2019 *vs*. 2022	0.7058	0.7058
U chromosome		
1995 *vs*. 2019	**0.0000**	**0.0000**
1995 *vs*. 2022	**0.0007**	**0.0011**
2019 *vs*. 2022	**0.0327**	**0.0327**
E chromosome		
1995 *vs*. 2019	0.2176	0.3264
1995 *vs*. 2022	0.9004	0.9004
2019 *vs*. 2022	0.0618	0.1854
O chromosome		
1995 *vs*. 2019	0.8561	0.8561
1995 *vs*. 2022	0.5899	0.8561
2019 *vs*. 2022	0.7839	0.8561

Table S5. Statistical comparisons between *CTI* values computed from Petnica population (1995, 2019, 2020, 2021 and 2022). In the rows, the values of the statistic test are presented and the corresponding adjusted *p* values are shown below in brackets. Significant values are in bold.

Year	1995	2019	2020	2021	2022
1995	–	1.925 (0.1427)	1.902 (0.1427)	1.207 (0.3251)	3.103 (**0.0190**)
2019		–	−0.107 (0.9147)	−1.061 (0.3463)	1.564 (0.1963)
2020			–	−1.012 (0.3463)	1.770 (0.1534)
2021				–	2..761 (**0.0290**)
2022					–

Table S6. CTI values from all Balkan populations. Populations are classified according to the climate. The information of year and month of each sample is also presented.

Population	Year	Month	CTI	Climate	Reference
Apatin	1994	June	−0.347	Cfa	[46]
Apatin	2008	June	−0.219	Cfa	[46]
Apatin	2009	June	−0.025	Cfa	[46]
Apatin	2018	June	0.342	Cfa	[46]
Kamariste	1996	June	0.258	Cfa	[42]
Petnica	1995	June	0.281	Cfa	[43]
Petnica	1995	May	0.122	Cfa	[43]
Petnica	1995	Aug	0.072	Cfa	[43]
Petnica	2010	May	0.354	Cfa	[43]
Petnica	2019	June	0.165	Cfa	Present research
Petnica	2020	June	0.170	Cfa	Present research
Petnica	2021	June	0.211	Cfa	Present research
Petnica	2022	June	0.097	Cfa	Present research
Zanjic	1997	June	0.205	Cfa	[42]
Avala	2003	Sept	0.385	Cfb	[40]
Avala	2004	June	0.374	Cfb	[40]
Avala	2004	Sept	0.333	Cfb	[40]
Avala	2005	Sept	0.207	Cfb	[40]
Avala	2011	June	0.426	Cfb	[40]
Avala	2014	June	0.383	Cfb	[40]
Avala	2015	July	0.262	Cfb	[40]
Avala	2016	June	0.302	Cfb	[40]
Avala	2017	June	0.371	Cfb	[40]
Djerdap	2001	Aug	0.343	Cfb	[45]
Djerdap	2001	June	0.162	Cfb	[45]
Djerdap	2002	June	0.299	Cfb	[45]
Fruska Gora	1971?	?	−0.062	Cfb	[55]
Jastrebac	1990	June	0.120	Cfb	[44]
Jastrebac	1993	June	0.151	Cfb	[44]

The symbol "?" stand for not known.

References

[40] Zivanovic, G.; Arenas, C.; Mestres, F. Adaptation of *Drosophila subobscura* chromosomal inversions to climatic variables: The Balkan natural population of Avala. *Genetica* **2021**, *149*, 155–169. https://doi.org/10.1007/s10709-021-00125-7.

[42] Zivanovic, G.; Andjelkovic, M.; Marinkovic, D. Chromosomal inversion polymorphism of *Drosophila subobscura* from southeastern part of Europe. *J. Zool. Syst. Evol. Res.* **2002**, *40*, 201–204.

[43] Zivanovic, G.; Arenas, C.; Mestres, F. Short- and long-term changes in chromosomal inversion polymorphism and global warming: *Drosophila subobscura* from the Balkans. *Isr. J. Ecol. Evol.* **2012**, *58*, 289–311. https://doi.org/10.1560/IJEE.58.4.289.

[44] Zivanovic, G.; Milanovic, M.; Andjelkovic, M. Chromosomal inversion polymorphism of *Drosophila subobscura* populations from Jastrebac Mountain shows temporal and habitat-related changes. *J. Zool. Syst. Evol. Res.* **1995**, *33*, 81–83. https://doi.org/10.1111/j.1439-0469.1995.tb00959.x.

[45] Zivanovic, G. Seasonal changes in chromosomal inversion polymorphism in a *Drosophila subobscura* natural population from a south-eastern European continental refugium of the last glaciation period. *Russ. J. Genet.* **2007**, *43*, 1344–1349. https://doi.org/10.1134/S1022795407120022.

[46] Zivanovic, G.; Arenas, C.; Mestres, F. Rate of change for the thermal adapted inversions in *Drosophila subobscura*. *Genetica* **2019**, *147*, 401–409. https://doi.org/10.1007/s10709-019-00078-y.

[55] Andjelkovic, M.; Sperlich, D. Inversion polymorphism in a Pannonian population of *Drosophila subobscura*. *Egypt. J. Genet. Cytol.* **1973**, *2*, 144–147.
